# Abnormalities in the genes that encode Large Amino Acid Transporters increase the risk of Autism Spectrum Disorder

**DOI:** 10.1002/mgg3.1036

**Published:** 2019-11-07

**Authors:** Lauren Cascio, Chin‐Fu Chen, Rini Pauly, Sujata Srikanth, Kelly Jones, Cindy D. Skinner, Roger E. Stevenson, Charles E. Schwartz, Luigi Boccuto

**Affiliations:** ^1^ JC Self research Institute Greenwood Genetic Center Greenwood SC USA

**Keywords:** amino acids, Autism spectrum disorder (ASD), large amino acid transporter (LAT), metabolism, *SLC7A5*

## Abstract

**Background:**

Autism spectrum disorder (ASD) is a common neurodevelopmental disorder whose molecular mechanisms are largely unknown. Several studies have shown an association between ASD and abnormalities in the metabolism of amino acids, specifically tryptophan and branched‐chain amino acids (BCAAs).

**Methods:**

Ninety‐seven patients with ASD were screened by Sanger sequencing the genes encoding the heavy (*SLC3A2*) and light subunits (*SLC7A5* and *SLC7A8*) of the large amino acid transporters (LAT) 1 and 2. LAT1 and 2 are responsible for the transportation of tryptophan and BCAA across the blood–brain barrier and are expressed both in blood and brain. Functional studies were performed employing the Biolog Phenotype Microarray Mammalian (PM‐M) technology to investigate the metabolic profiling in lymphoblastoid cell lines from 43 patients with ASD and 50 controls with particular focus on the amino acid substrates of LATs.

**Results:**

We detected nine likely pathogenic variants in 11 of 97 patients (11.3%): three in *SLC3A2*, three in *SLC7A5*, and three in *SLC7A8*. Six variants of unknown significance were detected in eight patients, two of which also carrying a likely pathogenic variant.

The functional studies showed a consistently reduced utilization of tryptophan, accompanied by evidence of reduced utilization of other large aromatic amino acids (LAAs), either alone or as part of a dipeptide.

**Conclusion:**

Coding variants in the LAT genes were detected in 17 of 97 patients with ASD (17.5%). Metabolic assays indicate that such abnormalities affect the utilization of certain amino acids, particularly tryptophan and other LAAs, with potential consequences on their transport across the blood barrier and their availability during brain development. Therefore, abnormalities in the LAT1 and two transporters are likely associated with an increased risk of developing ASD.

## Graphical abstract

Abnormalities in the metabolism of amino acids have been reported in cases with ASD. We detected 9 likely pathogenic variants in the genes encoding the subunits of the large amino acid transporters (LAT 1 and 2) in 11 of 97 patients (11.3%). Metabolic assays suggest that such variants may affect the utilization of certain amino acids, particularly tryptophan and other LAAs, with potential consequences on their transport across the blood barrier and their availability during brain development.

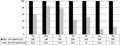

## 1 INTRODUCTION

Autism spectrum disorder (ASD) is a neurodevelopmental condition characterized by three core signs: impairment of social interactions, communication issues, and repetitive behaviors. Although the ASD incidence has recently risen to 1:59 school age children in the United States (Baio et al., 2018), the molecular mechanisms underlying this condition are still largely unknown. A series of studies have suggested a potential association between ASD and abnormalities in the metabolism of certain amino acids, such as tryptophan or branched‐chain amino acids (BCAAs) like leucine, isoleucine, and valine (Al‐Otaish et al., 2018; Anwar et al., 2018; Boccuto et al., 2013; Gevi, Zolla, Gabriele, & Persico, 2016; Kałużna‐Czaplińska, Jóźwik‐Pruska, Chirumbolo, & Bjørklund, 2017; Ming, Stein, Barnes, Rhodes, & Guo, 2012; Novarino et al., 2012; Smith et al., 2019; Tărlungeanu et al., 2016; West et al., 2014). The combined group of aromatic (tryptophan, phenylalanine, and tyrosine) and branch‐chained amino acids is defined as large neutral amino acids (LNAAs). Such amino acids play a key role in neurodevelopment and abnormalities in their metabolism may have critical consequences, both because of the perturbation of protein homeostasis (Louros & Osterweil, 2016) and their role as precursors of neuroactive molecules, such as serotonin (Muller, Anacker, & Veenstra‐VanderWeele, 2016).

Utilization of the Phenotype Mammalian MicroArray technology has demonstrated a reduced metabolic utilization of the amino acid tryptophan (Boccuto et al., 2013), which functions as a molecular precursor of neuroactive molecules associated with ASD, such as serotonin (Muller et al., 2016) and melatonin (Rossignol & Frye, 2014), key regulators of neurodevelopment, such as kynurenic acid and quinolinic acid, and the energy transporter nicotinamide adenine dinucleotide (NAD) (Stone & Darlington, 2002). Further analysis, employing stability selection, confirmed the abnormal metabolism of the amino acid tryptophan in ASD cells and indicated potential perturbations in the utilization of other large amino acids, such as tyrosine and the BCAAs (Hofner, Boccuto, & Göker, 2015).

A recent study by Tărlungeanu et al. reported two homozygous mutations (p.Ala246Val and p.Pro375Leu) in the *SLC7A5* gene in patients with ASD and motor delay, and described the impact of such mutations on amino acid transport across the blood‐brain barrier in a mouse model (Tărlungeanu et al., 2016). Significantly, reduced levels of leucine and isoleucine and increased levels of histidine were detected in the brains of *Slc7a5^−/−^* mice, having an autism‐related phenotype, that was rescued by intracerebroventricular injection of BCAAs. These findings suggest potential disruption of large amino acid transport in individuals with ASD. The large amino acid transporter (LAT) protein complexes 1 and 2 are expressed in both blood and brain, and include a constant heavy subunit, encoded by the *SLAC3A2* gene, and a light subunit that can be encoded by the *SLC7A5* gene (constituting LAT1) or by *SLC7A8* (LAT2).

In order to further investigate the role of LAT 1 and 2 in ASD, a genetic screening of the three genes encoding subunits of LAT 1 and 2 (*SLC3A2*, *SLC7A5*, and *SLC7A8*) was conducted in a cohort of 97 patients with ASD. In addition, metabolic profiles with particular focus on the utilization of large amino acids were analyzed in 12 of the 17 cell lines carrying sequence variants in the LAT genes.

## 2 MATERIALS AND METHODS

### 2.1 Patient and control cell lines

We screened genomic DNA from peripheral blood samples obtained from 97 patients, 90 with non‐syndromal ASD and 7 with syndromal ASD, including the 87 patients reported by Boccuto et al. (2013). Overall, 84 of these ASD patients were male and 13 were female (6.5:1 ratio). The age range was 2.5 to 34.25 years at the time the blood sample was obtained. The 90 patients with non‐syndromal ASD were part of the South Carolina Autism Project (SCAP). Eighty‐six of them were diagnosed with autistic disorder and four with pervasive developmental disorder not otherwise specified (PDD‐NOS), based on evaluation using the Autism Diagnostic Interview‐Revised (ADI‐R) and according to the Diagnostic and Statistical Manual of Mental Disorders (DSM) IV‐Revised criteria. The clinical and neurobehavioral features of these patients have been reported in a previous study (Schroer et al., 1998). Molecular tests excluded abnormalities in plasma amino acid levels, major chromosomal abnormalities, and pathogenic variants in genes associated with ASD: *ADNP*, *ALDH5A1*, *AMT*, *AP1S2*, *ARID1B*, *ARX*, *ATRX*, *BCKDK*, *BRAF*, *CACNA1C*, *CASK*, *CDKL5*, *CHD7*, *CHD8*, *CNTNAP2*, *CREBBP*, *CTNNB1*, *DHCR7*, *DYRK1A*, *EHMT1*, *FGD1*, *FMR1*, *FOLR1*, *FOXG1*, *FOXP1*, *FOXP2*, *GABRB3*, *SLC2A1*, *GRIN2B*, *HDAC8*, *HOXA1*, *HPRT1*, *KDM5C*, *KIRREL3*, *L1CAM*, *LAMC3*, *MBD5*, *MECP2*, *MED12*, *MEF2C*, *MID1*, *NHS*, *NIPBL*, *NLGN3*, *NLGN4X*, *NRXN1*, *NSD1*, *NTNG1*, *OPHN1*, *PAFAH1B1*, *PCDH19*, *PHF6*, *PNKP*, *PQBP1*, *PTCHD1*, *PTEN*, *PTPN11*, *RAB39B*, *RAD21*, *RAI1*, *RELN*, *SCN1A*, *SCN2A*, *SETBP1*, *SETD2*, *SHANK3*, *SLC9A6*, *SMC1A*, *SMC3*, *STXBP1*, *SYNE1*, *TBL1XR1*, *TBR1*, *TCF4*, *TMEM231*, *TMLHE*, *TSC1*, *TSC2*, *TUBA1A*, *UBE3A*, *UBE3C*, *VPS13B*, *ZEB2*. The seven syndromal patients were diagnosed with autistic disorder based on evaluation using the ADI‐R and the Autism Diagnostic Observation Schedule (ADOS), according to the DSM IV‐Revised criteria.

For the Biolog metabolic assays, lymphoblastoid cell lines from 43 patients with ASD, 12 with LAT variants and 31 without, were compared to cell lines from 50 age‐matched controls, which were matched by age range (1.2–10.3 years at blood sampling) and geographic area (Schroer et al., 1998). This control cohort was composed of 24 males and 26 females (ratio 0.92), since previous data indicated no major metabolic differences related to gender in control cell lines. No viable lymphoblastoid cells were available from the remaining five patients carrying sequence variants in the LAT genes.

Informed consent was approved by the Self Regional Healthcare Institutional Review Board (IRB) for Human Research and was reviewed and signed by all the participants and/or their legal guardians. The IRB approved the use of the samples in the studies reported in this paper.

Cell lines were obtained by immortalization of lymphocytes from blood samples using Epstein–Barr virus. The lymphoblastoid cell lines were harvested in Sigma RPMI‐1640 with 75 ml fetal bovine serum from Atlanta Biological (Flowery Branch) and 5 ml L‐Glutamine and 5 ml antibiotic/antimycotic from Sigma‐Aldrich (St. Louis).

### 2.2 Sanger sequencing

The *SLC3A2* gene is composed of 12 exons according to Ensembl genome browser, using sequence ENSG00000168003 (NC_000011.10 according to NCBI build GRCh38.p7). The *SLC7A5* gene is composed of 10 exons according to Ensembl genome browser, using sequence ENSG00000103257 (NC_000016.10 according to NCBI build GRCh38.p7). The *SLC7A8* gene is composed of 11 exons according to Ensembl genome browser, using sequence ENSG00000092068 (NC_000014.9 according to NCBI build GRCh38.p7). In order to amplify the coding regions and the intron/exon boundaries of these genes, the Primer 3 Input web tool [http://frodo.wi.mit.edu/primer3/] was used to design the primers utilized for gene sequencing (Supplemental File 1).

All variants identified by direct sequencing were analyzed using available bioinformatic websites. All substitutions were submitted to the following bioinformatic websites to assess their potentially deleterious effect on the proteins: Combined Annotation Dependent Depletion (CADD) website [http://cadd.gs.washington.edu/home], Sorting Tolerant from Intolerant (SIFT) [http://sift.bii.a-star.edu.sg/], PolyPhen‐2 [http://genetics.bwh.harvard.edu/pph2/index.shtml], Mutation Taster [http://www.mutationtaster.org/], and Mutation Assessor [http://mutationassessor.org/v1/]. All variants were also examined for splice sites that may have been created or deleted, using the Splice Site Calculator [http://192.168.50.223/splicing/maxent.cgi] and the Splice Site Score Calculation websites [http://rulai.cshl.edu/new_alt_exon_db2/HTML/score.html]. These alterations were also analyzed by RESCUE‐ESE [http://genes.mit.edu/burgelab/rescue-ese/] to see if the new variant introduced or removed an exon splicing enhancer (ESE) site. For the frequency of reported single nucleotide polymorphisms (SNPs) in the general population, the following browsers have been used as sources: Exome Aggregation Consortium (ExAC) [http://exac.broadinstitute.org/], genome Aggregation Database (gnomAD, using both exomic and genomic databases) [http://gnomad.broadinstitute.org/], and NHLBI Exome Sequencing Project (ESP) Exome Variant Server [http://evs.gs.washington.edu/EVS/].

### 2.3 Biolog metabolic arrays

The Phenotype Mammalian MicroArray (PM‐M) developed by Biolog (Hayward) is designed to measure the cellular production of NADH (nicotinamide adenine dinucleotide, reduced form) in the presence of different compounds. The methodology employs microplates with diverse carbon energy sources (plate PM‐M1), as well as amino acids, both alone and as dipeptides (plates PM‐M2 to M4). Each well contains a single chemical as the sole energy source and production of NADH per well is monitored using a colorimetric redox dye chemistry (Bochner et al., 2011). PM‐M plates were incubated with 20,000 lymphoblastoid cells per well in a volume of 50 μL, using the modified Biolog IF‐M1 medium. This medium was prepared by adding the following to 100 mL of Biolog IF‐M1: 1.1 mL of 100 × penicillin/streptomycin solution, 0.16 mL of 200 mM Glutamine (final concentration 0.3 mM), and 5.3 mL of fetal bovine serum (final concentration 5%). The cells were incubated for 48 hr at 37°C in 5% CO_2_. During the 48‐hr incubation, the only energy source available to the cells was the chemical in the well. After this first incubation, Biolog Redox Dye Mix MB was added (10 μL/well) and the plates were incubated under the same conditions for an additional 24 hr, during which time the cells metabolize the sole carbon source in the well. As the cells metabolize the carbon source, tetrazolium dye in the media is reduced, producing a purple color according to the amount of NADH generated.

At the end of the 24‐hr incubation, the plates were analyzed utilizing a microplate reader with readings at 590 and 750 nm. The first value (A_590_) indicated the highest absorbance peak of the redox dye and the second value (A_750_) gave a measure of the background noise. The relative absorbance (A_590‐750_) was calculated per well.

### 2.4 Data processing and statistical analysis of Phenotype Mammalian MicroArray (PM‐M) data

For PM‐M data, we collect the endpoint absorbance readings over 24 hr of incubation. The data from the tested patients with ASD (*n* = 43) and age‐matched controls (*n* = 50) are normalized using empty plates (*n* = 3) containing only the Biolog medium and dye without cells. The normalized absorbance readings are then transformed to a logarithmic scale. Although we acknowledge that differences in amino acid metabolism have been reported among patients with ASD (Smith et al., 2019), for the purpose of the analysis, we assumed that patients with ASD in our cohort shared as a group a similar profile in terms of NADH generation in the presence of amino acids as sole energy source. Our goal was to identify the wells in which patients with ASD were significantly different from the typically developing (TD) controls. We considered potential confounding factors that may have affect the metabolic or clinical profile of the patients in our cohort, including IQ scores, utilizing the non‐parametric Mann–Whitney's *t*‐test.

We utilized the program RStudio (version 1.1.456) to implement the non‐parametric Mann–Whitney's *t*‐test to determine differences in terms of significant wells between the ASD and TD samples. False discovery rates were controlled for multiple testing using the Benjamini and Hochberg technique of *p‐*value correction. The wells are considered significant if the adjusted *p‐*value is less than .05.

## 3 RESULTS

### 3.1 Sequencing of the genes encoding LAT subunits

Sanger sequencing of the coding regions and the intron/exon boundaries of the three genes encoding LAT subunits detected nine likely pathogenic variants in 11 out of 107 patients with ASD (10.3%) and another six variants of uncertain significance in 8 patients (7.5%).

**Figure 1:**
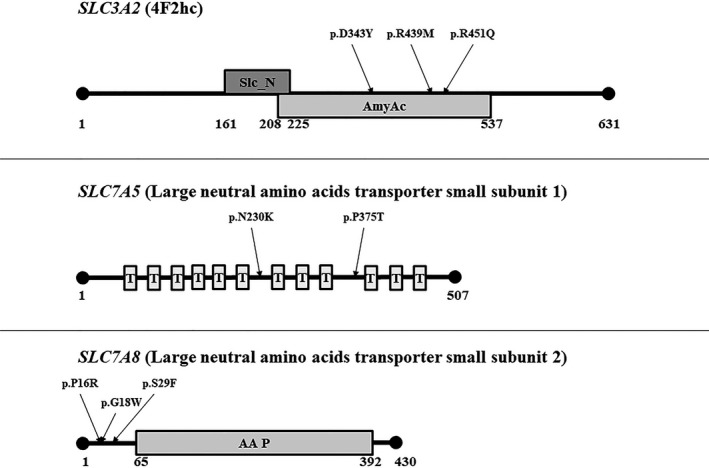
LAT protein domains and positions of the detected missense variants. Slc_N: solute carrier family 3 member 2 N‐terminus domain; AmyAc: alpha‐amylase catalytic domain; T: transmembrane domain; AA P: amino acid permease domain

In the *SLC3A2* gene four missense variants were detected in four unrelated patients. Additionally, three silent variants were detected in the cohort (Table 1). The *SLC3A2* gene encodes the 4F2 cell‐surface antigen heavy chain (hc) protein, a multifunctional type II membrane glycoprotein involved in amino acid transport and cell fusion, adhesion, and transformation (Figure 1). It constitutes the constant heavy subunit in both LAT1 and 2 (Mastroberardino et al., 1998). The bioinformatic predictions classified two of the missense variants as deleterious. One of them, c.1027G > T; p.Asp343Tyr (RefSeq NP_001012680.1), was not present in any database and was not detected in a cohort of 542 controls. The patient (18,454) also carries the p.Leu536Leu silent variant, which does not appear to produce any deleterious effect on the protein function. The other missense change, c.1352G > A; p.Arg451Gln (RefSeq NP_001012680.1), was found at a very low frequency in population databases (allele frequency ~ 0.0001–0.0002). Among the silent changes, the c.6G > A; p.Glu2Glu variant is predicted to create 4 new exon splicing enhancers (ESEs) and it is present at a very low frequency in the population databases (allele frequency 0.00006–0.0005). All detected missense variants fall within the alpha‐amylase catalytic domain (spanning residues 208 to 537), which is critical for the binding and transportation of the amino acids by LAT.

**Table 1:** Sequence variants detected in the *SLC3A2* gene

Patient	Variant	Bioinformatics	Databases
12,382	c.826G > A; p.Asp276Asn^a^	CADD: 0.002 (benign) SIFT: tolerated PolyPhen−2: benign Mutation taster: polymorphism Mutation Assessor: low impact	rs80190679 **ExAC (A): 0.0007** **gnomAD genome (A): 0.0006** **gnomAD exome (A): 0.0007** **NHLBI ESP (A): 0.0013**
**18,454**	**c.1027G > T; p.Asp343Tyr** a	**CADD: 25.6 (deleterious)** **SIFT: deleterious** **PolyPhen−2: possibly damaging** **Mutation Taster: disease causing** **Mutation Assessor: medium impact**	**No; 0/542 controls**
**17,843**	**c.1316G > T; p.Arg439Met** a	**CADD: 20.8 (deleterious)** SIFT: tolerated PolyPhen−2: benign **Mutation Taster: disease causing** **Mutation Assessor: medium impact**	rs116829615; **ExAC (A): 0.003** **gnomAD genome (A): 0.0018** **gnomAD exome (A): 0.0029** **NHLBI ESP (A): 0.0015**
**17,410**	**c.1352G > A; p.Arg451Gln** a	**CADD: 25.3 (deleterious)** **SIFT: deleterious** **PolyPhen−2: possibly damaging** **Mutation Taster: disease causing** **Mutation Assessor: medium impact**	**rs148443520** **ExAC (A): 0.0001** **gnomAD genome (A): 9.70E−05** **gnomAD exome (A): 0.0001** **NHLBI ESP (A): 0.0002**
17,550	c.6G > A; p.Glu2Glu^b^	**4 new ESEs**	rs114958414 **ExAC (A): 6.86E−05** **gnomAD genome (A): 0.0005** **gnomAD exome (A): 6.11E−05** **NHLBI ESP (A): 0.0005**
15,294	c.594T > G; p.Ala198Ala^a^	No change	rs114506587 **ExAC (A): 0.0017** **gnomAD genome (A): 0.0027** **gnomAD exome (A): 0.0008** **NHLBI ESP (A): 0.0027**
17226–9754–18454	c.1606C > T; p.Leu536Leu^a^	No change	rs76688902 **ExAC (A): 0.0072** **gnomAD genome (A): 0.0214** **gnomAD exome (A): 0.0057** **NHLBI ESP (A): 0.0253**

Abbreviation: ESE, Exon splicing enhancer.

**Bold**, likely pathogenic variant.

a Parents not available.

b *De novo*

A missense variant was detected in the *SLC7A5* gene affecting the same p.Pro375 residue (RefSeq NP_003477.4) reported by Tărlungeanu et al. (Tărlungeanu et al., 2016), although the alteration in this case (c.1123C > A, Table 2) causes a p.Pro375Thr change rather than p.Pro375Leu (c.1124C > T). The protein product of *SLC7A5* is large neutral amino acid transporter small subunit 1 (Figure 1), which functions as light subunit in LAT1 (Verrey, 2003). The p.Pro375 residue is highly conserved, de novo, not present in any database, not present in 542 controls, threonine is less similar to proline than leucine, and bioinformatic websites predict the change as being deleterious. Thus it is reasonable to infer that the functional impact of the p.Pro375Thr likely leads to variations in the substrate binding/translation activity and significantly reduced BCAA uptake, as assessed for the p.Pro375Leu (Tărlungeanu et al., 2016).

**Table 2:** Sequence variants detected in the *SLC7A5* gene

Patient	Variant	Bioinformatics	Databases
**14,988 Hom**; 9754–12388–12970 Het	**c.690C > G; p.Asn230Lys**	CADD: 7.151 (not deleterious) SIFT: tolerated PolyPhen−2: benign **Mutation Taster: disease causing** Mutation Assessor: neutral **Lost 2 ESEs**	rs1060250 **ExAC (G): 0.0046** **gnomAD genome (G): 0.0145** **gnomAD exome (G): 0.0035** **NHLBI ESP (G): 0.0175**
**10,471**	**c.1123C > A; p.Pro375Thr** a	**CADD: 25.1 (deleterious)** **SIFT: deleterious** **PolyPhen−2: probably damaging** **Mutation Taster: disease causing** **Mutation Assessor: medium risk**	**No; 0/542 controls**
**17,410**	**c.816−18C > T** a	**Abnormal quantile signal at acceptor splice site**	rs754179345; **0/542 controls** **ExAC (T): 1.67E−05** **gnomAD genome (T): 6.47E−05** **gnomAD exome (T): 1.63E−05** NHLBI ESP (G): no data
15,301	c.1290 + 44G>A^a^	**Lost 1 ESE**	rs774937771 ExAC (G): no data **gnomAD genome (G): 9.69E−05** **gnomAD exome (G): 8.43E−06** NHLBI ESP (G): no data

Abbreviations: Hom, homozygous; Het, heterozygous; ESE, Exon splicing enhancer.

**bold,** likely pathogenic variant.

a *De novo.*

The only homozygous variant of the study, c.690C > G; p.Asn230Lys (RefSeq NP_003477.4), was also detected in *SLC7A5* (Table 2). The variant is predicted to cause the loss of two exon splicing enhancer sites, and has been reported at a relatively low frequency in population databases: allele frequency 0.0035–0.0145, with 26 homozygous carriers out of 138,606 individuals (0.019%). The missense change was also present as a heterozygous variant in three patients, one of which, 9,754, also carries the c.1606C > T; p.Leu536Leu silent variant in *SLC3A2* (Table 1). A third potentially pathogenic variant, c.816‐18C > T, was detected in the *SLC7A5* gene. It was de novo and appears to affect the acceptor splice site in intron 4. It is reported at an extremely low frequency in the population databases (6 out of 275,840 alleles, 2.18E‐05), and was not detected in 542 controls.

Another alteration, c.1290 + 44G>A, was predicted to cause the loss of one ESE site in *SLC7A5* intron 8 and the minor allele frequency in the gnomAD database is between 8.43E‐06 and 9.69E‐05 (Table 2).

The *SLC7A8* gene encodes the large neutral amino acids transporter small subunit 2 (Figure 1), which binds the 4F2hc protein and functions as the light subunit in LAT2 (Pineda et al., 1999). Three missense variants were detected in this gene: p.Pro16Arg, p.Gly18Trp (de novo in both cases), and p.Ser29Phe (RefSeq NP_036376, Table 3). The variants were classified as deleterious by most of the bioinformatic websites and had relatively low frequencies in population databases (allele frequency range 0.0009–0.008). The patient with the p.Pro16Arg variant (12,388) also carries the c.690C > G; p.Asn230Lys change in *SLC7A5* (Table 2). Another de novo variant was detected in intron 7 of *SLC7A8*, c.1016‐49T > C. The change is not present in any population database and was not detected in 542 controls (Table 3).

**Table 3:** Sequence variants detected in the *SLC7A8* gene

Patient	Variant	Bioinformatics	Databases
**12,388**	**c.47C > G; p.Pro16Arg** a	**CADD: 18.69 (Deleterious)** SIFT: tolerated PolyPhen−2: benign Mutation taster: polymorphism Mutation Assessor: neutral	rs147920363 **ExAC (G): 0.001** **gnomAD genome (G): 0.0035** **gnomAD exome (G): 0.0009** **NHLBI ESP (G): 0.0032**
**6320–17673**	**c.52G > T; p.Gly18Trp** b	**CADD: 33 (Deleterious)** **SIFT: deleterious** **PolyPhen−2: possibly damaging** **Mutation Taster: disease causing** Mutation Assessor: low impact	rs144958980 **ExAC (T): 0.0017** **gnomAD genome (T): 0.0058** **gnomAD exome (T): 0.0014** **NHLBI ESP (T): 0.0058**
**4,947**	**c.86C > T; p.Ser29Phe** a	**CADD: 25.1 (Deleterious)** **SIFT: deleterious** PolyPhen−2: benign **Mutation Taster: disease causing** Mutation Assessor: low impact	rs149980964 **ExAC (T): 0.008** **gnomAD genome (T): 0.0019** **gnomAD exome (T): 0.0072** **NHLBI ESP (T): 0.0028**
15,318	c.1017−49T > C^b^	None	**No; 0/542 controls**

Note

**bold**, likely pathogenic variant.

a Parents not available

b *De novo*

### 3.2 Metabolic profiling of cell lines carrying LAT genes variants

Metabolic profiling data were obtained from lymphoblastoid cell lines from 43 patients with ASD and 50 controls with particular focus on the substrates of LATs (Table 4). Twelve of these patients have sequence variants or low expression of the LAT genes, while no genetic abnormalities were detected in the LAT genes in the remaining 31 patients (raw data in Supplemental File 2).

**Table 4:** Number of wells containing LNAAs and histidine with abnormal levels of NADH detected in patients with ASD and genetic variants in the LAT genes

Patient	Alteration	Trp	Phe	Tyr	Val	Ile	Leu	His
17,550	*SLC3A2* p.Glu2Glu	12/26 (46.1%)	**25/27 (92.6%)**	**25/27 (92.6%)**	*30/34 (88.2%)*	*21/25 (84%)*	**28/31 (90.3%)**	*19/22 (86.4%)*
12,382	*SLC3A2* p.Asp276Asn	**26/26 (100%)**	**27/27 (100%)**	**26/27 (96.3%)**	**34/34 (100%)**	**25/25 (100%)**	**31/31 (100%)**	**22/22 (100%)**
18,454	*SLC3A2* p.Asp343Tyr + p.Leu536Leu	**26/26 (100%)**	**27/27 (100%)**	**27/27 (100%)**	**34/34 (100%)**	**25/25 (100%)**	**30/31 (96.8%)**	**22/22 (100%)**
17410^a^	*SLC3A2* p.Arg451Gln + *SLC7A5* c.816−18C > T	**15/15 (100%)**	6/15 (40%)	*14/17 (82.3%)*	*16/22 (72.7%)*	1/3 (33.3%)	3/6 (50%)	*2/3 (66.7%)*
17,226	*SLC3A2* p.Leu536Leu	**26/26 (100%)**	**27/27 (100%)**	**27/27 (100%)**	**34/34 (100%)**	**25/25 (100%)**	**31/31 (100%)**	**22/22 (100%)**
15,294	*SLC3A2* p.Ala198Ala	**26/26 (100%)**	**27/27 (100%)**	**27/27 (100%)**	**34/34 (100%)**	**24/25 (96%)**	**31/31 (100%)**	**22/22 (100%)**
10,471	*SLC7A5* p.Pro375Thr	**26/26 (100%)**	*20/27 (74.1%)*	**26/27 (96.3%)**	*26/34 (76.5%)*	*18/25 (72%)*	*22/31 (71%)*	*14/22 (63.6%)*
15,301	*SLC7A5* c.1290 + 44G>A	**26/26 (100%)**	**27/27 (100%)**	**25/27 (92.6%)**	**33/34 (97.1%)**	**25/25 (100%)**	**30/31 (96.8%)**	*18/22 (81.8%)*
12,970	*SLC7A5* p.Asn230Lys	5/26 (19.2%) 13/26 (50%) H	9/27 (33.3%) 11/27 (40.7%) H	3/27 (11.1%) 16/27 (59.3%) H	3/34 (8.8%) *22/34 (64.7%) H*	13/25 (52%) 3/25 (12%) H	3/31 (9.7%) 15/31 (48.4%) H	**21/22 (95.5%) H**
14,988	*SLC7A5* p.Asn230Lys Hom	**26/26 (100%) H**	**27/27 (100%) H**	**26/27 (96.3%) H**	**34/34 (100%) H**	**25/25 (100%) H**	**31/31 (100%) H**	**22/22 (100%) H**
4,947	*SLC7A8* p.Ser29Phe	**26/26 (100%)**	**27/27 (100%)**	**27/27 (100%)**	**34/34 (100%)**	**24/25 (96%)**	**31/31 (100%)**	**22/22 (100%)**
15,318	*SLC7A8* c.1016−49T > C	**25/26 (96.2%)**	**27/27 (100%)**	**26/27 (96.3%)**	**33/34 (97.1%)**	**25/25 (100%)**	**31/31 (100%)**	**20/22 (90.9%)**

Note

**Bold**, >90% of wells showing significant differences between patients and controls.

*Italics*, 60%–89% of wells showing significant differences between patients and controls.

a These cell lines were only tested on PM‐M4 plates; H: the levels of NADH are significantly higher in patients with ASD than in controls, in all other wells the levels in patients are lower.

The metabolic findings confirmed a reduced production of NADH in the wells containing tryptophan and the other aromatic amino acids revealed similar trends: 10/12 (83.3%) samples showed reduced NADH levels in most of the wells containing tyrosine and 9/12 (75%) in the ones containing phenylalanine (Table 4). Decreased energetic metabolism was also consistently detected in at least 70% of the wells containing BCAAs and specifically in 10 cell lines (83.3%) for valine, and in 9 (75%) for isoleucine and leucine (Table 4). The data for histidine were slightly less consistent: 8 cell lines (66.7%) showed reduced generation of NADH in at least 70% of the wells containing this amino acid (Table 4).

The only cell line carrying the homozygous variant c.690C > G; p.Asn230Lys (14,988) revealed increased utilization of the amino acids analyzed in all but one well. Another cell line heterozygous for the same variant (12,970) showed both reduced and increased utilization of LNAAs and increased utilization of histidine.

**Figure 2:**
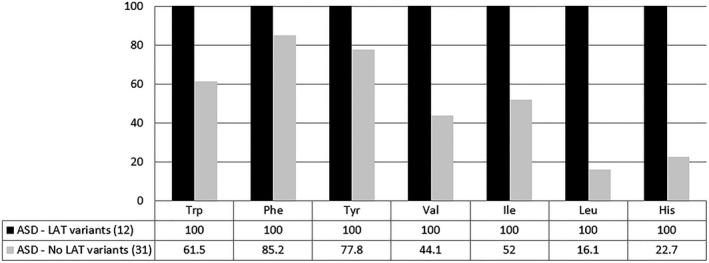
Utilization of LAT substrates in patients with ASD with and without variants in LAT genes. Graphic representation of the percentage of wells containing LNAAs (tryptophan, phenylalanine, tyrosine, valine, isoleucine, and leucine) and histidine showing significantly lower levels of NADH as compared to 50 control cell lines. Black: cell lines from patients with ASD with sequence variants or abnormal expression levels in LAT genes. Grey: cell lines from patients with ASD without abnormal findings in LAT genes. The percentages are based on endpoint absorbance raw data calculated on the two cohorts collectively. See Supplemental Files 3 and 4 for more details

**Figure 3:**
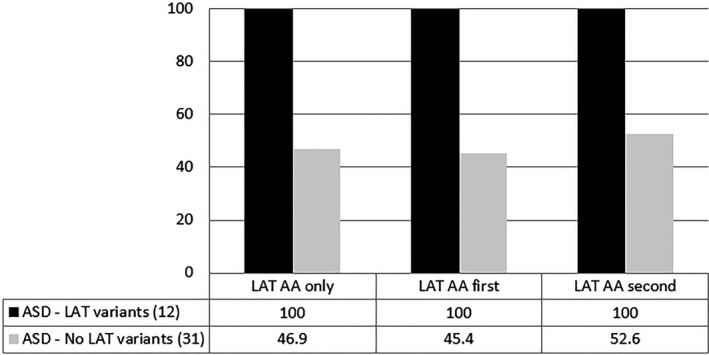
Utilization of LAT substrates according to their distribution in the arrays. Graphic representation of the percentage of wells containing amino acids related to LAT1 and 2 (LAT AA) showing significantly lower levels of NADH as compared to 50 control cell lines. LAT AA only: wells containing either LAT‐related amino acids alone or dipeptides constituted exclusively by LAT‐related amino acids. LAT AA first: wells containing dipeptides with a LAT‐related amino acid in the first position. LAT AA second: wells containing dipeptides with a LAT‐related amino acid in the second position. Black: cell lines from patients with ASD with sequence variants or abnormal expression levels in LAT genes. Grey: cell lines from patients with ASD without abnormal findings in LAT genes. The percentages are based on endpoint absorbance raw data calculated on the two cohorts collectively. See Supplemental Files 3 and 4 for more details

A statistical analysis of the collective data comparing the 12 cases with LAT findings and the 31 without findings showed a significantly reduced generation of NADH in more than half of the wells containing aromatic amino acids in cases without LAT findings, albeit not in as many wells as in the cases with abnormalities in the LAT genes (Figure 2). A drastic reduction of significantly different wells was instead noticed for isoleucine, leucine, valine, and histidine, suggesting that the abnormalities detected in the LAT genes in this study may have a more relevant impact on the metabolism of BCAAs and histidine. The molecular form of the amino acid did not seem to affect the metabolic response, since a clear difference between cases with and without LAT abnormalities has been observed for wells containing only LAT‐related amino acids, or with a LAT‐related amino acid in the first or second position of dipeptides (Figure 3).

## 4 DISCUSSION

Abnormalities in the metabolism of large amino acids have been reported in patients with ASD (Al‐Otaish et al., 2018; Anwar et al., 2018; Boccuto et al., 2013; Gevi et al., 2016; Kałużna‐Czaplińska et al., 2017; Ming et al., 2012; Novarino et al., 2012; Tărlungeanu et al., 2016; West et al., 2014), suggesting a potential predisposing role for gene variants affecting the structure and function of the two main channels transporting large amino acids, LAT1 and 2. Homozygous variants in the *SLC7A5* gene, encoding the light subunit of LAT1, have previously been proven to impair amino acid transport at the blood–brain barrier in mice leading to neurological and behavioral abnormalities associated with ASD (Tărlungeanu et al., 2016). In this study, nine likely pathogenic variants in the genes encoding the LAT subunits, *SLC3A2*, *SLC7A5*, and *SLC7A8*, were detected in 11 of the 97 patients with ASD (11.3%). Another six variants of uncertain significance were found and were not considered deleterious according to bioinformatic predictions and frequency in control databases. Six variants, two of which likely pathogenic, were de novo in seven patients, while for eight variants no parental samples were available. Therefore, coding variants in the LAT genes were overall detected in 17 patients with ASD (17.5%). Metabolic profiling utilizing the Biolog platform was performed in cell lines from 12 of these 17 patients and showed abnormal utilization of large neutral amino acids in all of them.

All 17 patients carrying sequence variants in the LAT genes were diagnosed with non‐syndromal ASD. No differences have been noted in the clinical presentation of patients with and without abnormalities in the LAT genes. IQ scores were collected on 11 of the 12 patients with LAT abnormalities and metabolic assays and ranged from 15 to 68, with an average of 40.2, falling within the range of moderate intellectual disability (IQ = 36–51), while in the 31 cases without LAT abnormalities the IQ ranged from 20 to 93 (average 43.9) (*p* = .9785).

Four variants, three of which are likely pathogenic, were not present in population databases and were not detected in 542 controls. All variants were heterozygous, except for the p.Asn230Lys substitution in *SLC7A5*, which was heterozygous in three cases and homozygous in one individual (14,988). This was also the most recurrent variant in the cohort, followed by the *SLC3A2* p.Leu536Leu (three cases) and by the *SLC7A8* p.Gly18Trp (two cases). In two of the three patients carrying the *SLC3A2* p.Leu536Leu silent change, another variant in a LAT gene was detected: the *SLC3A2* p.Asp343Tyr in patient 18,454 (Table 1) and the *SLC7A5* p.Asn230Lys in 9,754 (Table 2). In another individual (12,388) two changes were detected: the *SLC7A8* p.Pro16Arg and the *SLC7A5* p.Asn230Lys.

Although no mutation hotspots were identified, some variants seem to cluster in certain domains of the proteins encoded by the three LAT genes. All four missense variants in *SLC3A2* fall within the alpha‐amylase catalytic domain (Figure 1), which is critical for the binding and transportation of the amino acids (Mastroberardino et al., 1998). The changes in *SLC7A8* are clustered in 14 residues (16 to 29) and are located at the N‐terminus of the protein, before the amino acid permease 2 domain starting at residue 65 (Figure 1). The findings suggest that the potential impact of these variants should not affect directly the transportation of the amino acid substrates, but rather they may influence the protein stability and proper localization on the cellular membrane.

Lymphoblastoid cell lines from 12 patients with sequence variants in the LAT genes were tested using the Biolog metabolic arrays to assess the amino acid transportation and utilization as energy source (Table 4). All cell lines showed a generalized reduction in the production of NADH in the presence of large neutral amino acids (alone or as part of dipeptides) as energy sources. Another 31 cell lines from patients without findings in the LAT genes were tested for comparison versus the same 50 controls and showed an overall less significant reduction in the production of NADH in the presence of LNAAs and histidine, as compared to the 12 cell lines with LAT abnormalities (Figure 2). The analysis of the metabolic response to individual amino acids indicated moderate differences in the wells containing aromatic amino acids and more drastic ones in the wells containing BCAAs and histidine. These findings suggest that the abnormalities in the LAT genes reported in this study may affect the metabolism of branch‐chained amino acids and histidine more than aromatic amino acids, which is in line with the observations by Tărlungeanu et al. (2016). Possible explanations for this discrepancy are the sites and types of the variants, that might affect the binding affinity for smaller amino acids only, and the possible compensation for the cellular intake of aromatic amino acids operated by alternative transporters.

Consistently reduced NADH levels in wells containing all the large neutral amino acids were noted in four patients with variants in the *SLC3A2* gene, 12,382 (pAsp276Asn), 18,454 (p.Asp343Tyr + p.Leu536Leu), 17,226 (p.Leu536Leu), and 15,294 (p.Ala198Ala), and in two with *SLC7A8* variants, 4,947 (p.Ser29Phe) and 15,318 (c.1016‐49T > C). However, no particular genotype–phenotype correlation was noted for the gene or the domain affected by the variant, the type of variant (exonic vs. intronic, missense vs. silent), or the presence of one or more variants in the LAT genes in the same patient (see Table 4).

The only cell line carrying a homozygous change, the *SLC7A5* p.Asn230Lys, was also the only sample showing an increased utilization of all large neutral amino acids as compared to controls (Table 4), indicating perhaps a gain‐of‐function effect on the LAT1 complex that may cause increased binding affinity of the transporter for its substrates. A cell line heterozygous for the same variant (12,970) showed increased NADH levels in some of the wells containing LNAAs and in all but one containing histidine, while a reduction of NADH production was observed only in some wells containing isoleucine, phenylalanine, and tryptophan (see Table 4 and Supplemental File 2). These data suggest that augmented utilization of large amino acids as energy sources may still generate a metabolic perturbation by increasing the intracellular levels of amino acids and reducing their availability outside of the cells and in the blood circulation. They also suggest that the amount of large amino acids crossing the cell membrane requires a fine regulation and that both deficient and excessive transportation may be deleterious for the cell metabolism.

Overall, the findings indicate that certain heterozygous variants in the LAT genes are sufficient to cause perturbation of the transportation of aromatic amino acids, BCAAs, and, to a lesser extent, histidine. A previous microarray expression study (Boccuto et al., 2013) showed that reduced transcript levels of the *SLC7A5* and *SLC7A8* genes was associated with reduced utilization of tryptophan in lymphoblastoid cell lines from 10 individuals with ASD, suggesting that reduced expression of LAT genes may affect large amino acids metabolism in ASD as well. As observed in previous studies (Boccuto et al., 2013; Hofner et al., 2015), metabolic utilization of other amino acids does not seem to be significantly affected in cell lines from patients with ASD (Supplemental Files 3 and 4).

While homozygous mutations in the *SLC7A5* gene have been reported in seven patients from two consanguineous families presenting with ASD and motor delay (Tărlungeanu et al., 2016), heterozygous variants in the same gene, and in one case even in the same p.P375 residue, as well as in the other two LAT genes (*SLC3A2* and *SLC7A8*) appear to be associated with increased risk of non‐syndromal ASD. In vivo mouse data also showed an increased brain levels of histidine, which crosses the blood–brain barrier via antiportal transportation with large neutral amino acids through LAT1 (Tărlungeanu et al., 2016). The data generated in this study do not replicate the increased cellular intake of histidine, except for a few samples. A possible interpretation for this discrepancy is that the effect of heterozygous variants may influence the interaction of the transporters with large neutral amino acids other than histidine, which also binds specific transporters for basic amino acids. Moreover, the type of amino acid transporters expressed in lymphoblastoid cells and in the blood–brain barrier is slightly different and may have different binding affinity for histidine. It must be considered that the metabolic arrays employed in this work are not the ideal assays to test membrane transporters’ activity or amino acid uptake. However, they were utilized in order to correlate the previous observations on NADH production in the presence of amino acids (Boccuto et al., 2013; Hofner et al., 2015) with genetic variants in the LAT genes. Future studies will be necessary to further investigate such correlation employing transporter‐specific assays.

Recent works focused on sequencing of large populations of patients with ASD have not reported pathogenic variants in the LAT genes (Coe et al., 2019; Guo et al., 2018; SPARK Consortium, 2018), probably because of the lack of evidence of potentially deleterious effects of heterozygous variants in *SLC3A2*, *SLC7A5*, and *SLC7A8* in patients with ASD. The data generated by this study suggest that such variants affect cellular energetic metabolism by disrupting amino acid transport and seem to be associated with ASD.

Metabolic assays indicate that even silent changes in *SLC3A2* or variants in *SLC7A5* or *SLC7A8* not considered as pathogenic by bioinformatic predictors are associated with abnormal production of NADH in the presence of large neutral amino acids in cells from individuals with ASD. All the six variants of uncertain significance reported are either extremely rare or not present in population databases, either de novo or detected in patients for which no parental samples were available, and in two of them a potential impact on exon splicing enhancer has been predicted in silico (Tables 1, 2, 3). Therefore, it is plausible that these variants may have some effect on the protein expression and/or function. In order to further investigate the impact of these variants on the LAT function, other functional studies will be performed, including transporter assays assessing substrate uptake.

## 5 CONCLUSIONS

A wide range of sequence variants in the LAT genes can affect the utilization of certain amino acids, even if they do not alter directly the protein sequence. The most affected amino acids appear to be tryptophan and BCAAs, whose abnormal metabolism has been previously reported in patients with ASD (Boccuto et al., 2013; Gevi et al., 2016; Kałużna‐Czaplińska et al., 2017; Novarino et al., 2012; Tărlungeanu et al., 2016). The abnormal function of LAT1 or two complexes may have potential repercussions on the transportation of these amino acids across the blood–brain barrier and their availability during brain development. Therefore, abnormalities in the LAT1 and two transporters appear to affect of the cell utilization of large neutral amino acids, leading to decreased availability of these amino acid and eventually imbalance of the protein homeostasis, which has been associated with an increased risk of developing ASD.

## CONFLICT OF INTEREST.

The authors declare no conflict of interest.

## AUTHOR CONTRIBUTIONS.

Lauren Cascio and Sujata Srikanth have performed the experiments; Chin‐Fu Chen and Rini Pauly have performed the statistical analysis; Kelly Jones has managed the cell cultures; Cindy Skinner has coordinated the patients’ enrollment; Roger Stevenson has coordinated the clinical and genetic evaluation of the patients; Charles Schwartz has supervised the experiments and the writing of the manuscript; Luigi Boccuto has supervised the experiments, interpreted the data, and written the manuscript.
