# Supplementary material for: Abnormalities in the genes that encode Large Amino Acid Transporters increase the risk of Autism Spectrum Disorder
Source: Mol Genet Genomic Med. 2019 Nov 7;8(1):e1036. doi: 10.1002/mgg3.1036 (PMC6978257; doi:10.1002/mgg3.1036)
Supplement: Supplementary file 1 [file MGG3-8-e1036-s001.pdf]

Supplemental files.

Table S1. Primer sequences for LAT genes.

SLC3A2	
SLC3A2 M13-1 F	gtaaaacgacggccagCCCTCTAACCCTGTTCTGAGC
SLC3A2 M13-1 R	caggaaacagctatgacTGACATACTTTCCCGAAATGG
SLC3A2 M13-2new F	gtaaaacgacggccagAGAGTTGGGGTCTCACTGTGT
SLC3A2 M13-2new R	caggaaacagctatgacATTACCTATCATCTCGGCATTCTG
SLC3A2 M13-3 F	gtaaaacgacggccagCCACTGTGCCCAACCATACT
SLC3A2 M13-3 R	caggaaacagctatgacAAAGACAAATTCTACCAGATAGCAAG
SLC3A2 M13-4 F	gtaaaacgacggccagGGTCCAGGTAGGGGTTGAG
SLC3A2 M13-4 R	caggaaacagctatgacCTAGATCCGTCTGGGGACAG
SLC3A2 M13-5 F	gtaaaacgacggccagCTTAGGCGCTGGGAGAAGG
SLC3A2 M13-5 R	caggaaacagctatgacAAGTTCTAGCCTACTTTTCCTCTGG
SLC3A2 M13-6 F	gtaaaacgacggccagTGACTTGCATTTGTGATTTCCT
SLC3A2 M13-6 R	caggaaacagctatgacTCTACAGGGCTTGCTGTGAA
SLC3A2 M13-7-8 F	gtaaaacgacggccagGAGGTTTAGTGTGGGCTGGA
SLC3A2 M13-7-8 R	caggaaacagctatgacAGGCCTAGCACTCCACTGAG
SLC3A2 M13-9-10 F	gtaaaacgacggccagCTGGCCCCATTCTTTCTTGT
SLC3A2 M13-9-10 R	caggaaacagctatgacGCCATCCCCAGAATCATCA
SLC3A2 M13-11-12 F	gtaaaacgacggccagACAGATGTGAGCCACCATGC
SLC3A2 M13-11-12 R	caggaaacagctatgacAGGGCCTGGGAAGGAAAG
SLC7A5	
SLC7A5-Ex1-new-M13-F	gtaaaacgacggccagGCCGAGGAGAAGGAAGAGG
SLC7A5-Ex1-new-M13-R	caggaaacagctatgacTACTCACGCACGCAGAGG
SLC7A5 M13-2 F	gtaaaacgacggccagCTGAGCTGGCGTCTGTCTG
SLC7A5 M13-2 R	caggaaacagctatgacTGAAGGACACACAGGCAAAG
SLC7A5 M13-3 F	gtaaaacgacggccagGACCTCTGTTCAGCGCTCTC
SLC7A5 M13-3 R	caggaaacagctatgacTGGACACGTCAGGGACTGTA
SLC7A5 M13-4 F	gtaaaacgacggccagCGGTGATTTATTTTCCCTGA
SLC7A5 M13-4 R	caggaaacagctatgacCTGCTTCCTGCCTCACATTA
SLC7A5 M13-5 F	gtaaaacgacggccagCACAGGGTTTGGCCTTCA
SLC7A5 M13-5 R	caggaaacagctatgacCTGGCCACAGCCTCTCAG
SLC7A5 M13-6 F new	gtaaaacgacggccagCCAGCTCACTGGTTGTGC
SLC7A5 M13-6 R	caggaaacagctatgacATGTTGAACCTGGCCATGAG
SLC7A5 M13-7 F	gtaaaacgacggccagCCAGAGAGGCCCAGACAAG
SLC7A5 M13-7 R	caggaaacagctatgacCTGTGGCAGCCTCCCTCT
SLC7A5 M13-8 F	gtaaaacgacggccagGACCTTGCATGTTGCTCAGA
SLC7A5 M13-8 R	caggaaacagctatgacCTCCCAACTCAGGGTCCTT
SLC7A5 M13-9 F	gtaaaacgacggccagGGTGACCTGGAAGTCAGCAG
SLC7A5 M13-9 R	caggaaacagctatgacGACGCCTCTCAACTCCCTTC

SLC7A5 M13-10 F	gtaaaacgacggccagCAGCCCCCAGGCTAACAC
SLC7A5 M13-10 R	caggaaacagctatgacCACAGCAGCCTCCACTGC
SLC7A8	
SLC7A8 M13-1 F	gtaaaacgacggccagAATTCAAAGCTCTCTCCCAAT
SLC7A8 M13-1 R	caggaaacagctatgacATCTCACAGGAGGACCACCA
SLC7A8 M13-2 F	gtaaaacgacggccagGAAATGAGACCACCCTCCAA
SLC7A8 M13-2 R	caggaaacagctatgacTGGCTTTTTCCAATCTGACC
SLC7A8 M13-3 F	gtaaaacgacggccagCTCTCCCCTGATGGCTCTG
SLC7A8 M13-3 R	caggaaacagctatgacCGCTCCAGCCTGAGTGAC
SLC7A8 M13-4 F	gtaaaacgacggccagGCATCATGCAGCCAGATACA
SLC7A8 M13-4 R	caggaaacagctatgacTACATGCAAGGGGAGGAAAG
SLC7A8 M13-5 F	gtaaaacgacggccagGGCCCCTGACTTGTCTCAC
SLC7A8 M13-5 R	caggaaacagctatgacATGGACACCTGCTCCCTCTT
SLC7A8 M13-5new F	gtaaaacgacggccagAGTTGGTGTCATTGCCACAG
SLC7A8 M13-5new R	caggaaacagctatgacACGGCAAATGGTTTAAGTGC
SLC7A8 M13-6 F	gtaaaacgacggccagCCTGGACTCCATGGCAAG
SLC7A8 M13-6 R	caggaaacagctatgacCAGGCAAGTCTATGTCTGCATT
SLC7A8 M13-7 F	gtaaaacgacggccagGAGTGAAGTGGGAGCTGAGG
SLC7A8 M13-7 R	caggaaacagctatgacCAAAGTGCACAGCAGAGTGG
SLC7A8 M13-8 F	gtaaaacgacggccagGCAGAAGGTAGGCAAGGATG
SLC7A8 M13-8 R	caggaaacagctatgacGAGAGGGAGGTGTGTGGAGA
SLC7A8 M13-9 F	gtaaaacgacggccagGAATATCTTTCGGATCACTGATGA
SLC7A8 M13-9 R	caggaaacagctatgacTGCAGCTAGAACCATAGAGGAA
SLC7A8 M13-10 F	gtaaaacgacggccagAGAATGCCCCAGTCCCTAGT
SLC7A8 M13-10 R	caggaaacagctatgacCTAGATCTCCTGGCCCTGGT
SLC7A8 M13-11 F	gtaaaacgacggccagCCAGGTTGGTGGGATACTCA
SLC7A8 M13-11 R	caggaaacagctatgacCAAGGCAGGGAGGTAGGATA
